# Fine Physical and Genetic Mapping of Powdery Mildew Resistance Gene *MlIW172* Originating from Wild Emmer (*Triticum dicoccoides*)

**DOI:** 10.1371/journal.pone.0100160

**Published:** 2014-06-23

**Authors:** Shuhong Ouyang, Dong Zhang, Jun Han, Xiaojie Zhao, Yu Cui, Wei Song, Naxin Huo, Yong Liang, Jingzhong Xie, Zhenzhong Wang, Qiuhong Wu, Yong-Xing Chen, Ping Lu, De-Yun Zhang, Lili Wang, Hua Sun, Tsomin Yang, Gabriel Keeble-Gagnere, Rudi Appels, Jaroslav Doležel, Hong-Qing Ling, Mingcheng Luo, Yongqiang Gu, Qixin Sun, Zhiyong Liu

**Affiliations:** 1 State Key Laboratory for Agrobiotechnology/Beijing Key Laboratory of Crop Genetic Improvement/Key Laboratory of Crop Heterosis Research & Utilization, Ministry of Education, China Agricultural University, Beijing, China; 2 Agriculture University of Beijing, Beijing, China; 3 Maize Research Center, Beijing Academy of Agricultural and Forestry Sciences, Beijing, China; 4 USDA-ARS West Regional Research Center, Albany, California, United States of America; 5 State Key Laboratory of Plant Cell and Chromosome Engineering, Institutes of Genetics & Developmental Biology, Chinese Academy of Sciences, Beijing, China; 6 Murdoch University, Perth, Western Australia, Australia; 7 Institute of Experimental Botany, Centre of Plant Structural and Functional Genomics, Olomouc, Czech Republic; 8 Department of Plant Sciences, University of California, Davis, Davis, California, United States of America; Nanjing Forestry University, China

## Abstract

Powdery mildew, caused by *Blumeria graminis* f. sp. *tritici*, is one of the most important wheat diseases in the world. In this study, a single dominant powdery mildew resistance gene *MlIW172* was identified in the IW172 wild emmer accession and mapped to the distal region of chromosome arm 7AL (bin7AL-16-0.86-0.90) via molecular marker analysis. *MlIW172* was closely linked with the RFLP probe *Xpsr680*-derived STS marker *Xmag2185* and the EST markers *BE405531* and *BE637476*. This suggested that *MlIW172* might be allelic to the *Pm1* locus or a new locus closely linked to *Pm1*. By screening genomic BAC library of durum wheat cv. Langdon and 7AL-specific BAC library of hexaploid wheat cv. Chinese Spring, and after analyzing genome scaffolds of *Triticum urartu* containing the marker sequences, additional markers were developed to construct a fine genetic linkage map on the *MlIW172* locus region and to delineate the resistance gene within a 0.48 cM interval. Comparative genetics analyses using ESTs and RFLP probe sequences flanking the *MlIW172* region against other grass species revealed a general co-linearity in this region with the orthologous genomic regions of rice chromosome 6, *Brachypodium* chromosome 1, and sorghum chromosome 10. However, orthologous resistance gene-like RGA sequences were only present in wheat and *Brachypodium*. The BAC contigs and sequence scaffolds that we have developed provide a framework for the physical mapping and map-based cloning of *MlIW172*.

## Introduction

Wheat accounts for approximately 30% of the global cereal consumption (FAO: World Agriculture: towards 2015/2030), and is of fundamental importance for food security. Ensuring the yield increase of wheat to meet future needs has become an important focus in agricultural research. Powdery mildew, caused by *Blumeria graminis* f. sp. *tritici* (*Bgt*), is one of the most devastating diseases of common wheat (*Triticum aestivum*, AABBDD; 2n = 6x = 42) in China and worldwide. Significant reductions of yield, flour quality, and other related grain qualities were reported when severe epidemics occurred in cool humid climates [1]. Development of resistant cultivars containing single or stacked resistance genes is a major focus of wheat breeding program because growth of such cultivars has proved to be the most effective agronomic approach to control disease losses. Currently, more than 60 powdery mildew resistance genes/alleles have been identified at 43 loci (*Pm1 – Pm50, Pm18 = Pm1c, Pm22 = Pm1e, Pm23 = Pm4c, Pm31 = Pm21*) in wheat and its wild relatives [2]–[4].

However, since major resistance genes tend to become ineffective within a short period due to rapid evolution of mildew populations, it is necessary to search continually for new sources of resistance in wheat breeding. Wild emmer, *T. turgidum* var. *dicoccoides* [*T. dicoccoides*, (AABB; 2n = 4x = 28)], as the progenitor of the cultivated tetraploid and hexaploid wheat, is crossable with both durum and common wheat and has great potential for wheat improvement [5]. Wild emmer is a valuable source of powdery mildew resistance [6]–[9] and has been extensively studied for identification of new alleles and genes useful for wheat improvement. Among the characterized wheat powdery mildew genes, *Pm16*, *Pm26*, *Pm30, Pm36*, *Pm41, Pm42, MlIW72*, *MlZec1, PmG3M, MlIW170, PmG16, PmAS846* and *HSM1* have been identified in wild emmer and introduced into cultivated wheat [3], [10].

Molecular marker technology has greatly accelerated gene/trait tagging, thereby improving development of elite variety through marker-assisted selection in breeding programs. Valuable genetic and genomic resources useful for molecular marker development in wheat are publicly available, and a total of 1,286,372 wheat expressed sequence tags (ESTs) have been deposited in the NCBI database (http://www.ncbi.nlm.nih.gov/). More than 16,000 ESTs have been mapped in the wheat deletion bins collection [11]. These resources provide opportunities for development of functional molecular markers [eg. sequence tagged sites (STS) and single nucleotide polymorphisms (SNP)], and performing comparative genomics analyses. Simple sequence repeat (SSR) and STS markers developed from ESTs are often associated with the coding regions of the genome and can be converted into easy and reliable PCR-based markers useful for trait mapping and marker assisted selection [12]–[14].

Although the complete genome sequence of wheat is not expected to be available in the near future due to the complexity and huge genome size, a large amount of wheat sequences have been generated to provide genome-wide sequence information for marker development [15]–[18]. In addition, the gene order in grass species was generally conserved [19]–[22] and the synteny facilitates comparative genomics analyses in grass families [23]. The availability of genome sequence information from rice [24], *Brachypodium* [25], and Sorghum [20] allows for improved comparisons and predictions of gene conservation in other genomes like wheat. The assumption is that if the gene order within a defined region is conserved across these three species (orthologous), the corresponding genomic region in wheat might have maintained similar gene conservation during evolution [15], [26]–[28]. These predictions enabled colinearity or synteny analyses, which served as a primary source of genome information for wheat marker development and mapping [29]–[31], [21].

In this paper, we report the identification of a powdery mildew resistance gene *MlIW172* derived from wild emmer and mapping the gene to chromosome arm 7AL. We have also developed a high-resolution genetic linkage map with alignment to a draft physical map covering the *MlIW172* region by using a combinational approach of comparative and genetic analysis, and BAC screening and sequencing.

## Materials and Methods

### Plant materials

Wild emmer accession IW172 (original accession No. G-797-M, originally provided by Dr. Z. Gerechter-Amitai of the Agricultural Research Organization, the Volcani Center, Israel), was highly resistant to *Bgt* isolate E09, a prevailing pathotype in Beijing, China, with infection type (IT) 0, in both the seedling and adult plant stages [32]. Durum wheat line Mo75 was highly susceptible to E09 with IT 3–4. The F_1_ hybrid between Mo75 and IW172 (11 F_1_ hybrids for initial genetic mapping and 127 F_1_ hybrids for fine mapping) was self-pollinated to generate an F_2_ segregating population and corresponding F_2:3_ families.

Three nulli-tetrasomics (N7AT7B, N7BT7A, and N7DT7A), two ditelosomics (DT7AS and DT7AL) and six 7AL deletion lines of hexaploid wheat Chinese Spring, (kindly provided by Drs. WJ Raupp and BS Gill, Wheat Genetics Resource Centre, Kansas State University, USA), were used for chromosome-arm assignment and bin mapping of molecular markers linked to the powdery mildew resistance locus since some markers were mapped on more than one chromosome before (GrainGenes, http://wheat.pw.usda.gov/GG2/index.shtml).

### Powdery mildew assessments

The prevailing *Bgt* isolate E09 used for powdery mildew evaluation was obtained from Dr. Xiayu Duan, Institute of Plant Protection, Chinese Academy of Agricultural Sciences, Beijing, China. Isolate E09 is virulent on *Pm1a*, *Pm3a*, *Pm3c, Pm5a*, *Pm6*, *Pm7*, *Pm8*, *Pm17* and *Pm19* [7], but avirulent on IW172. The E09 isolate was used to test the resistance of Mo75, IW172, F_1_, F_2_ plants and F_2:3_ progenies at the seedling stage under controlled greenhouse conditions. The F_2_-derived F_3_ families (20 seedlings of each F_3_ family) were tested to confirm the phenotypes and establish the resistance genotype of each F_2_ plant. Seedlings were inoculated with E09 when the first leaf was fully expanded. Inoculations were performed by brushing conidia from sporulating highly susceptible seedlings of common wheat cv. Xuezao. Infection types were scored 15 days after inoculation when the susceptible Xuezao control became heavily infected. Disease symptoms were recorded on scales of 0, 0;, and 1, 2, 3, 4, with 0 for no visible symptoms, 0; for necrotic flecks, and 1, 2, 3, 4 for highly resistant, resistant, susceptible and highly susceptible reactions, respectively. F_2_ genotypes were predicted based on the responses of the F_3_ families and classified as homozygous resistant, segregating and homozygous susceptible.

### Genomic DNA extraction and Bulked Segregant Analysis

Genomic DNA was extracted by the cetyltrimethylammonium bromide (CTAB) method [33] from uninfected seedlings of parental IW172 wild emmer, durum wheat Mo75 and F_2_ plants of the Mo75/IW172 cross. Equal amounts of DNA from 10 homozygous resistant and 10 homozygous susceptible individuals of each F_3_ progeny were randomly selected to establish resistant and susceptible DNA pools for bulked segregant analysis (BSA) [34]. Each bulk was at a final concentration of 50 ng·µl^−1^. The DNA concentration for BSA was measured using Quant-iT PicoGreen dsDNA Assay Kit (Invitrogen).

### Molecular marker analysis

Initially, 175 SSR primer pairs (*Xgwm, Xwmc, Xbarc*, and *Xcfa* series in GrainGenes 2.0 website http://wheat.pw.usda.gov/GG2/index.shtml), mapped to A and B genomes of wheat were chosen to screen the parents, resistant and susceptible DNA bulks. The resulting polymorphic markers were used to genotype the F_2_ population. After that, the Chinese Spring nulli-tetrasomics and deletion stocks of homoeologous group 7 were used to determine the chromosomal and bin locations of these polymorphic makers. In addition, STS markers closely linked to the *Mlm2033* and *Mlm80* powdery mildew resistance genes on chromosome arm 7AL were used for analysis [35]. Polymerase chain reaction (PCR) was conducted in 10 µl reactions containing 10 mM Tris-HCl, pH 8.3, 50 mM KCl, 1.5 mM MgCl_2_, 0.2 mM dNTPs, 25 ng of each primer, 50 ng of genomic DNA, and 0.75 U of *Taq* DNA polymerase, and DNA amplifications were conducted at 94°C for 5 min, followed by 40 cycles at 94°C for 45 s, 50–60°C (depending on specific primers) for 45 s, and 72°C for 90 s, and the reactions were terminated after a final extension at 72°C for 10 min. The PCR products were mixed with 2 µl of loading buffer (98% formamide, 10 mM EDTA, 0.25% bromophenol blue, and 0.25% xylene cyanol), separated on 8% non-denaturing polyacrylamide gels (39 acrylamide: 1 bisacrylamide), and visualized following silver staining.

### Comparative genomics analysis and EST-STS marker development

To saturate the region containing the *MlIW172* resistance gene with molecular markers, sequences of RFLP probes PSR121, PSR148, PSR680, MWG2062, and CDO347 [36], [37] and 7AL bin mapped ESTs BE637476, BE405531 (MAG1757) and CD452874 (MAG1759) [35], [38] were used in Blastn searches against the genome sequences of rice, sorghum and *Brachypodium*. The corresponding syntenic genomic regions of rice, sorghum, and *Brachypodium* were identified for homology comparisons (Table 1). The complete set of rice genes [39] was downloaded from the “Rice Annotation Project” website at http://rice.plantbiology.msu.edu/index.shtml; *Brachypodium* and sorghum genes were obtained from “Phytozome” at http://www.phytozome.net/ [40]. To identify additional wheat EST-STS markers that are potentially linked to the resistance gene, the sequences of putative *Brachypodium* genes at the syntenic genomic region were used as queries to search for homologous wheat ESTs and these wheat ESTs were used to design PCR primers (Table 2) using primer premier 5.0 (http://www.premierbiosoft.com/primerdesign/). PCR products were separated on 8% non-denaturing PAGE gels [41] for polymorphism detections and polymorphic markers were tested on DNAs of F_2_ mapping population.

**Table 1:** Mapped wheat EST markers and ortholgous gene pairs among *Brachypodium*, rice and sorghum

Wheat maker	Wheat EST	Rice	*Brachypodium*	Sorghum	Pfam Description
*XRGA-C6 and XRGA-B6*	*TC432233*	*Os02g16060*	*Bradi1g29670*	*Sb04g009750*	NBS-LRR disease resistance protein
*NA*	*TC421430*	*Os06g51260*	*Bradi1g29680*	*Sb10g030960*	MYB family transcription factor
*NA*	*CA694329*	*Os06g51250*	*Bradi1g29690*	*Sb10g030940*	EF hand family protein
*NA*	*TC443015*	*Os06g51240*	*Bradi1g29710*	*Sb10g030930*	Expressed protein
*WGGC4655*	*CK153650*	*Os06g51220*	*Bradi1g29730*	*Sb10g030910*	HMG1/2
*WGGC4654*	*TC386221*	*Os09g15480*	*Bradi1g29750*	*Sb10g030900*	Ser/Thr-rich protein T10 in DGCR region
*WGGC4653*	*TC390087*	*Os06g51210*	*Bradi1g29760*	*Sb10g030890*	Zinc finger family protein, putative
*Xmag1759*	*CD452874*	*Os06g51170*	*Bradi1g29780*	*Sb10g030880*	Serine/threonine-protein kinase Cx32
*Xmag1757*	*BE405531*	*Os06g51160*	*Bradi1g29780*	*Sb10g030870*	Glycosyl transferase family 8
*BE637476*	*BE637476*	*Os06g51150*	*Bradi1g29800*	*Sb10g030840*	Catalase isozyme B

**Table 2:** EST-STS, EST-SSR and SSR markers linked to powdery mildew resistance gene *MlIW172*

Makers	Maker type	Forward primer (5′-3′)	Reverse primer (5′-3′)
*XRGA-B6*	EST-STS	TTGCTCTGCTCTTCTTCCTT	TATGGTGGTTGGTGGTATGT
*XRGA-C6*	EST-STS	ATTGGGACGGGGATGAAGAT	GGGCAGACAGGGAAAAAGTG
*WGGC4653*	EST-STS	ATCCATCACTTCACGCTC	GTTCCTAACCCAACAATGT
*WGGC4654*	EST-STS	AGACTAATGACTGACACGACG	CTGAAAGAACTGCTGTGC
*WGGC4655*	EST-STS	CATCCGCCTTCTTCGTCT	TTTCCGATTCGCTCAAGC
*WGGC4656*	EST-STS	GAGAGTGTTGGTTGTAGG	GCGAAGCATTTCCAGTAG
*WGGC4657*	EST-STS	TAATGTTGCTCACTTCCG	CCTCTTCCATAATGCGAT
*WGGC4658*	SSR	ACAGCGGCTTGTTTCTTG	CACTTGTCAGCATTTCATCC
*WGGC4659*	SSR	CATATCATGGTTGTCCTCCTA	TCTCAACTGAATTCGAAACAT
*WGGC4660*	SSR	TTAGCGTCATGTGATTAGGAT	CTAGAGGTCCCCAACATTATT
*WGGC4661*	SSR	CGACAATCATTTGTGTATGTG	TGTAGACACATTGTTGAAAGAAA
*WGGC4662*	SSR	CCAAGAGAAACGGATACAAAT	TTTACCTTGCAGATTTCTGTT
*WGGC4663*	EST-SSR	GAAAAAGAAAAACCAGGAGAA	GGGAACCAGTACACTAAACAC
*WGGC4664*	SSR	ATTGAAAAACGTGAAACCAG	CTCTGTTTGATCTGAGCGTAG
*WGGC4665*	SSR	GGAGCCAGTACACTAAACACA	AGGAGAAAACCGAGTAAAAAT
*WGGC4666*	SSR	CTCCGAAAATATTCAAATCAG	TTGGCATATGAACCAATACAT
*WGGC4667*	SSR	GAGGGAACCGTACAAATACA	ACTCACAGAGCTCACCAGAT
*WGGC4668*	SSR	GTTTGATCTGAGCGTAGGAC	AACGTGAAACCAGGTACAAC

### Data analysis

Deviations of observed data from theoretically expected segregation ratios were tested using Chi-squared (χ^2^) tests for goodness-of-fit. MapMaker 3.0 [42] was used to determine linkage of polymorphic markers and the resistance gene. A LOD score of 3.0 was used as the threshold for declaration of linkage. The genetic maps were constructed with the software Mapdraw V2.1 [43].

### Identification, sequencing and analysis of BAC clones

The *T. turgidum durum* cv. Langdon BAC library (including 516,096 BAC clones stored in 1,344 384-well plates) [44] was arrayed in 280 DNA pools and used for PCR screening with the co-segregated marker *XRGA-C6* based on the initial mapping result. Two positive BAC clones were identified. The Chinese Spring 7AL-specific BAC library (TaaCs7ALhA, 15.3×coverage, Keeble-Gagnere, manuscript in preparation) consists of 5,223 minimum tilling path (MTP) BAC clones was screened by PCR using the most closely linked marker *WGGC4663*. A BAC contig with 12 BACs was identified and selected for sequencing.

The two Langdon BACs' DNA was pooled together and the 12 Chinese Spring BACs' DNA was divided into 3 pools. The 4 pools were barcoded separately for 454 sequencing. Five micrograms of pooled BACs DNA was used to prepare the 454 sequencing library using the GS Titanium rapid library and 3kb span paired-end library preparation kit following the manufacturer's instructions (Roche Diagnostics). The 454 sequencing rapid libraries were processed using the GS FLX plus Titanium LV emPCR (Lib-L) and GS FLX plus Titanium sequencing (GS FLX+) kits (Roche Diagnostics) according to the manufacturer's instructions. The paired-end libraries were processed using GS FLX plus Titanium LV emPCR (Lib-L) and GS Titanium Sequencing Kit XLR70 (Roche Diagnostics). The cross-match program (University of Washington, Seattle, WA, USA) and Roche 454 Newbler were applied to remove the vector and barcode sequences and perform sequence assembly.

All analyses were performed on Linux systems. Gene prediction was performed using the combination of MAKER from GMOD (http://www.gmod.org/wiki/MAKER) and TriAnnot pipeline from URGI database (http://wheat-urgi.versailles.inra.fr/Tools). The NBS-LRR domain of resistance gene analogs (RGAs) was identified using Pfam (http://pfam.sanger.ac.uk/) and stand-alone BLAST obtained from the National Center for Biotechnology Information (NCBI: www.ncbi.nlm.nih.gov/). We used coding sequence data sets from the sorghum genome version 1 [20], rice (*Oryza sativa*) genome 6 (rice.plantbiology.msu.edu), and *Brachypodium* genome version 1 [25].

## Results

### Genetic analysis of the powdery mildew resistance gene in IW172

The IW172 wild emmer accession was highly resistant to the E09 *Bgt* isolate (IT value 0), whereas durum wheat line Mo75 was highly susceptible (IT value 4). The F_1_ plants from the Mo75× IW172 cross were highly resistant (IT value 0-0;), indicating complete dominance of resistance. In our initial genetic mapping using 115 F_2:3_ families, the observed ratio of 26 homozygous resistant: 55 segregating: 34 homozygous susceptible fitted the expected 1∶2∶1 ratio for monogenic resistance (χ^2^ = 1.33, lower than χ^2^_0.05_,_2_ = 5.99, 0.75>P>0.5; Table 3). In our fine mapping using a larger population of 4192 F_2:3_ families, the phenotypic segregation ratio was: 1046 homozygous resistant, 2159 segregating and 987 homozygous susceptible, and also corresponded to the expected 1∶2∶1 ratio for monogenic resistance (χ^2^ = 5.45, lower than χ^2^_0.05_,_2_ = 5.99, 0.1>P>0.05; Table 3). Hence, the powdery mildew resistance gene in IW172 was provisionally designated *MlIW172*.

**Table 3:** Genetic analysis of the powdery mildew resistance gene *MlIW172*

Population	Size	A	H	B	χ^2^	P	χ^2^_0.05_
**IW172**	20	20					
**Mo75**	20			20			
**Mo75/IW172**	20		20				
**F_2:3_**	115	26	55	34	1.33	0.5–0.75	5.99
**F_2:3_**	4192	1046	2159	987	5.45	0.05–0.1	5.99

A, H, B represent homozygous resistant, heterozygous resistant and homozygous susceptible, respectively.

### SSR and EST mapping of *MlIW172*

Bulked segregant analysis was employed to screen wheat *Xgwm* SSR primer pairs [45]. One marker, *Xgwm344* was polymorphic between the resistant and susceptible pools and confirmed to be linked to *MlIW172* in the F_2_ population. Since *Xgwm344* is closely linked to *Pm1e* on the distal part of 7AL [46], additional SSR markers on 7AL arm were screened for polymorphisms between the pools. Three SSR markers, *Xwmc525*, *Xcfa2040* and *Xcfa2240*, were found to be linked to the *MlIW172*.

**Figure 1:**
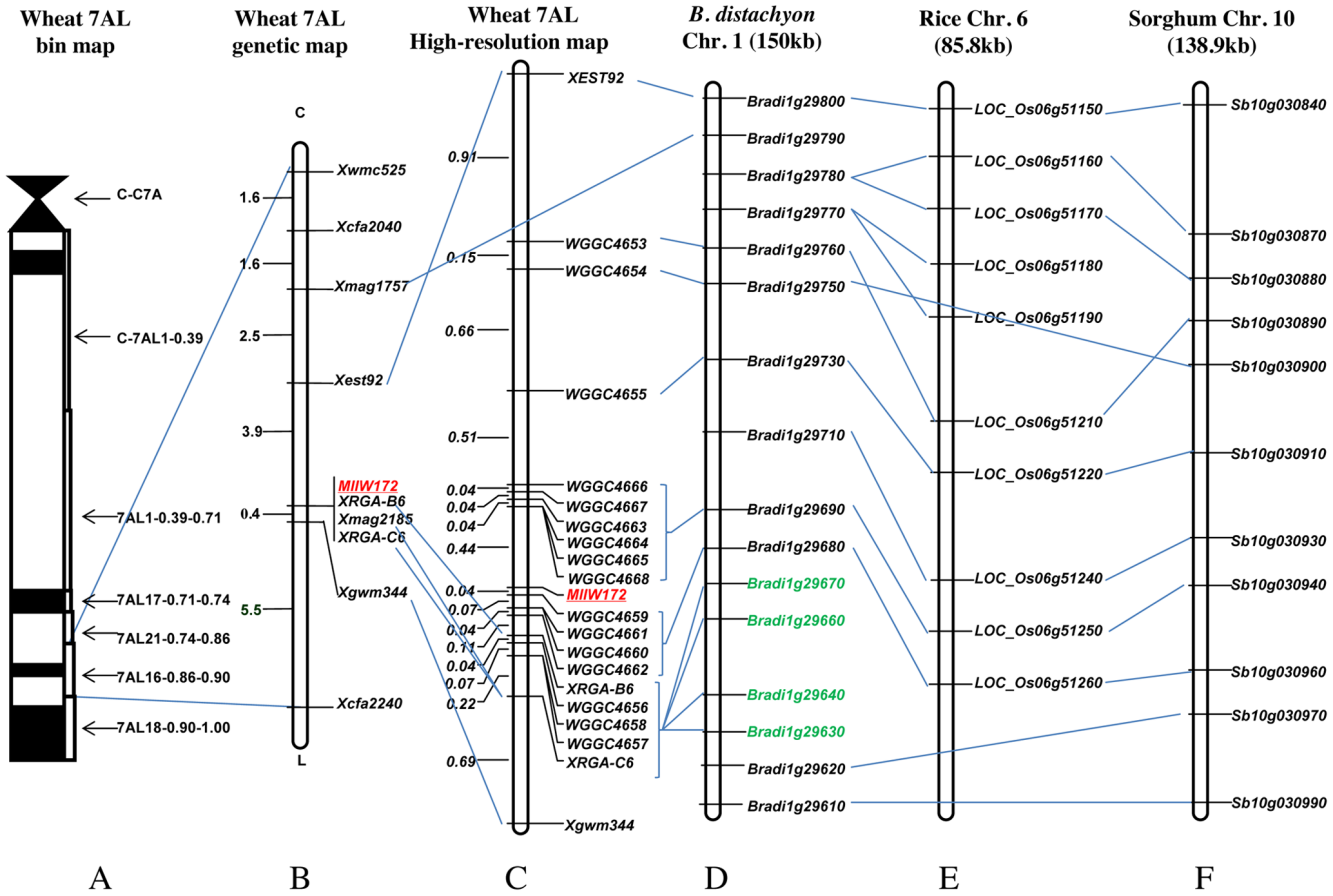
Genetic and comparative genomics linkage map of powdery mildew resistance gene *MlIW172* derived from wild emmer. **A:** *MlIW172* physical bin map. *MlIW172* was mapped to the distal bin 7AL16-0.86-0.90. **B:** Preliminary *MlIW172* genetic map on wheat chromosome arm 7AL with genetic distances in cM shown on the left, markers shown on the right. **C:** *MlIW172* high-resolution genetic map on wheat 7AL arm with genetic distances in cM shown on the left, EST-STS, EST-SSR and SSR markers shown on the right. Molecular markers that were previously assigned to the 7A wheat deletion bin map (**A**) are connected to the physical map with solid lines. The *MlIW172* locus is in red and underlined. The markers which served as anchors, establishing colinearity between the *MlIW172* genetic map and the sequences of *Brachypodium*, rice and sorghum, are connected to the *Brachypodium* gene with solid lines. **D:** The *MlIW172* orthologous genomic region on *Brachypodium* chromosome 1 (150kb) with orthologous genes shown on the right. The four genes in green represent the RGA cluster. **E:** The *MlIW172* orthologous genomic region on rice chromosome 6 (85.8kb) with orthologous genes shown on the right. **F:** The *MlIW172* orthologous genomic region on sorghum chromosome 10 (138.9kb) with orthologous genes shown on the right.

A set of Chinese Spring homoeologous group 7 nullisomic-tetrasomic, ditelosomic and deletion lines were employed for chromosomal bin assignment of *MlIW172-* linked SSR markers *Xgwm344*, *Xwmc525*, *Xcfa2240* and *Xcfa2040*. All four SSR markers were physically mapped to the distal bin 7AL-16 (0.86–0.90) (Fig. 1A), demonstrating that *MlIW172* maps to the distal part of 7AL.

EST-STS primer pairs that mapped physically to the distal 7AL-16 (0.86–0.90) bin [47] (http://wheat.pw.usda.gov/SNP/primers/contig_primer_list.xls) were selected to screen for polymorphisms between the IW172 and Mo75 parental lines, and the resistant and susceptible DNA pools. Out of 22 primer pairs tested, only one EST marker *BE637476* was polymorphic and linked with *MlIW172*. Five STS markers linked to the *Mlm2033* and *Mlm80* powdery mildew resistance genes located on chromosome 7AL [35] were also tested for polymorphism in the *MlIW172* mapping population. *MAG1757* and *MAG2185* were polymorphic between IW172 and Mo75, as well as the resistant and susceptible DNA pools and also linked to the *MlIW172* locus. Based on its linkage map position, *MlIW172* could be assigned to the corresponding chromosome region of the *Mlm2033*, *Mlm80* and *Pm1* loci on 7AL (Fig. 1B).

### Comparative mapping of the *MlIW172* region

In order to saturate the *MlIW172* genetic map, sequences of five RFLP probes, PSR121, PSR148, PSR680 (*MAG2185*), CDO347 and C607 [37] closely linked with *Pm1a*, and three ESTs, BE637476, BE405531 (*MAG1757*), and CD452874 (*MAG1759*) flanking *MlIW172* on the distal bin of 7AL were used as queries to search the rice, sorghum and *Brachypodium* genome sequences to identify syntenic genomic regions corresponding to the *MlIW172* locus in wheat using an expected value of 1E-10 and identity≧80% as cutoff points [48]. Both PSR148 and BE637476 detected putative orthologs on *Brachypodium* chromosome 1 (*Bradi1g29800*), rice chromosome 6 (*Os06g51150*) and sorghum chromosome 10 (*Sb10g030840*). A putative ortholog of BE405531 (*MAG1757*) was also found in *Brachypodium* (*Bradi1g29790*), but not in rice or sorghum. CD452874 (*MAG1759*) was homologous to the *Brachypodium* gene *Bradi1g29780*, which corresponded to two genes in rice (*Os06g51160* and *Os06g51170*) and sorghum (*Sb10g030870* and *Sb10g030880*), indicating gene duplications in rice and sorghum. PSR680 (*MAG2185*) was homologous to a RGA cluster (*Bradi1g29630*, *Bradi1g29640*, *Bradi1g29660*, and *Bradi1g29670*) in *Brachypodium* (Fig. 1D). However, no RGA was detected in the corresponding genomic regions of rice and sorghum (Fig. 1E, 1F). Nevertheless, our analysis revealed a syntenic relationship between the *MlIW172* genomic region in wheat chromosomal bin C-7AL16-0.86-0.90 (from BE637476 to *MAG2185*) and *Brachypodium* chromosome 1 (a 150-kb region from *Bradi1g29610* to *Bradi1g29800*), rice chromosome 6 (an 85.8-kb region from *Os06g51260* to *Os06g51150*) and sorghum chromosome 10 (a 138.9-kb region from *Sb10g030990* to *Sb10g030840*) (Table 2, Fig. 1D, 1E, 1F).

To accurately characterize collinearity between the *MlIW172* region and the corresponding genomic regions of *Brachypodium*, rice and sorghum, the sequences of putative *Brachypodium* genes from *Bradi1g29630* to *Bradi1g29800* were used as queries to search for orthologous wheat ESTs (http://wheat.pw.usda.gov/GG2/blast.shtml). A total of 58 wheat ESTs were identified and used to design primers with the Conserved Primers 2.0 [49]. Out of 76 primer pairs screened, 5 EST-STS makers, *XRGA-C6*, *XRGA-B6*, *WGGC4653*, *WGGC4654*, and *WGGC4655* (Table 2) were found to be polymorphic between the parental lines IW172 and Mo75, as well as the resistant and susceptible bulks, and were subsequently used to construct a *MlIW172* high-density linkage map after genotyping the recombinants identified by BE637476 and *Xgwm344* in the mapping population. *XRGA-C6* and *XRGA-B6* were derived from wheat ESTs orthologous to *Bradi1g29670*. The *WGGC4653*, *WGGC4654*, and *WGGC4655* markers were derived from three wheat ESTs that were orthologous to *Bradi1g29760*, *Bradi1g29750*, and *Bradi1g29730* in *Brachypodium* (Table 1). The orders of these markers were highly conserved between wheat, *Brachypodium*, rice, and sorghum (Fig. 1E).

### Marker enrichment using BAC libraries from cv. Langdon, Chinese Spring 7AL arm and *T. urartu* scaffolds

**Figure 2:**
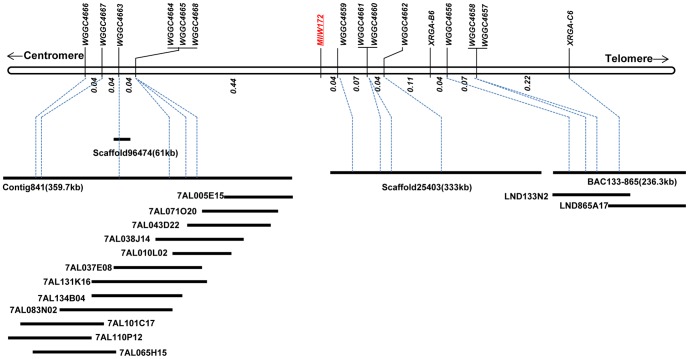
Physical map of the BAC contigs and scaffolds flanking the *MlIW172* locus anchored to the high-resolution genetic map. The approximate physical locations of all the newly designed markers are given on the BAC contigs or scaffolds.

**Figure 3:**
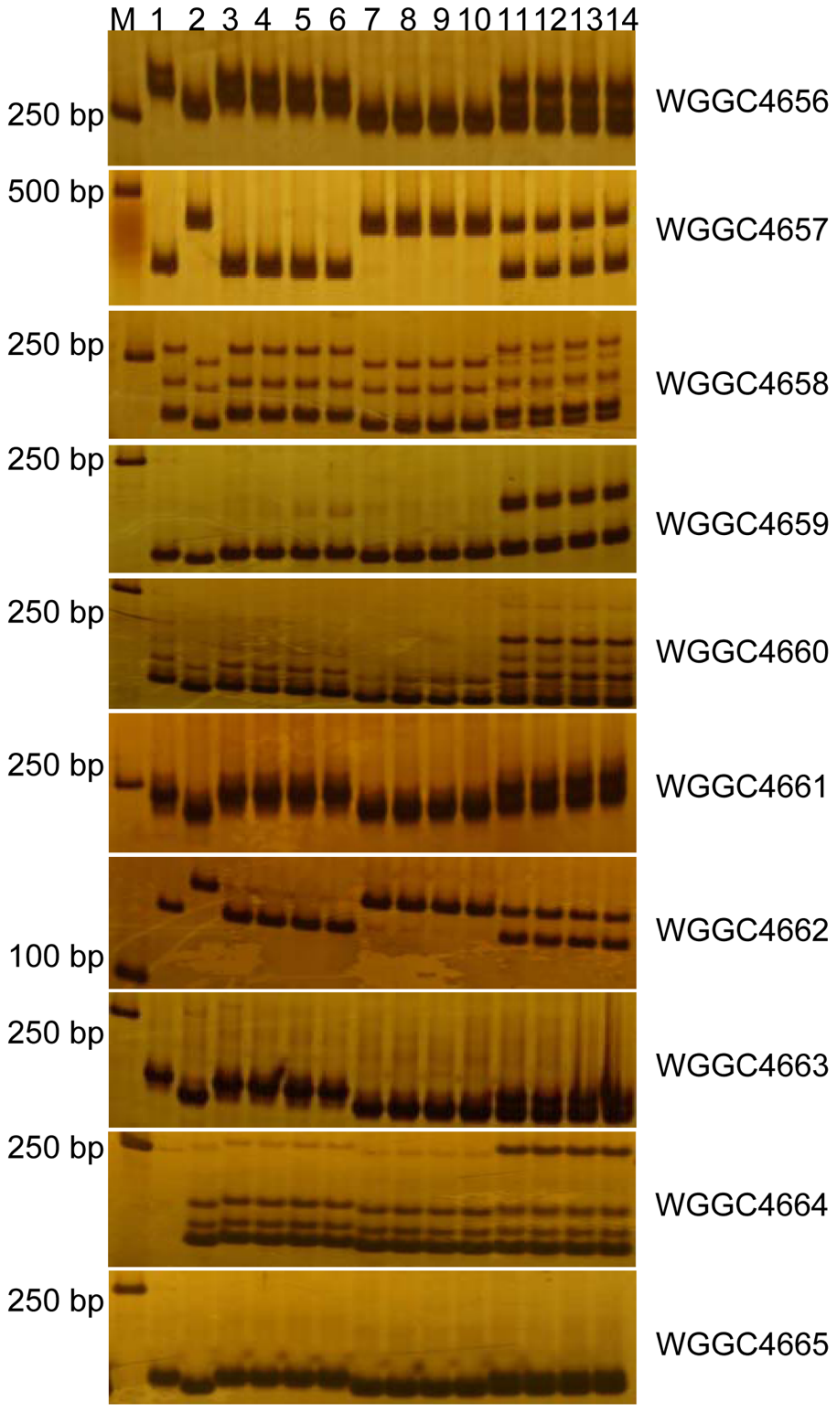
PCR amplification patterns of the markers *WGGC4656, WGGC4657, WGGC4658, WGGC4659, WGGC4660, WGGC4661, WGGC4662, WGGC4663, WGGC4664*, and *WGGC4665* in 8% non-denatured polyacrylamide gels. M: 2kb DNA marker. Lanes 1 and 2 are IW172 and Mo75, respectively, lanes 3–6 represent homozygous resistant plants, lanes 7–10 represent homozygous susceptible plants, and lanes 11–14 represent heterozygous resistant plants.

BAC libraries screening was employed to develop a physical map covering the *MlIW172* region and generate BAC sequences for marker development. However, BAC library from the IW172 wild emmer accession with the resistant trait is not yet available. We therefore screened BAC library of tetrapolid wheat cv. Langdon with the *XRGA-C6* marker and detected two BACs 133N2 and 865A17 (Fig. 2), which are 115 and 151 kb, respectively. Alignments of 454 sequences of these two BACs indicated they shared a 30 kb overlapping region and hence form a sequence contig spanning about 236.3 kb (GenBank accession No. KJ782374; Fig. 2). We developed two intron-flanking EST-PCR markers [50] *WGGC4656* and *WGGC4657* and one SSR marker *WGGC4658* from the contig to construct a higher resolution map (Table 2, Fig. 2, Fig. 3), and found that *WGGC4657* was co-segregated with *WGGC4658*.

To provide additional markers for higher resolution map, sequences of *Bradi1g29680*, *Bradi1g29690*, *Bradi1g29700*, *Bradi1g29710*, and *Bradi1g29720* were used as queries to blast the *T. urartu* genome scaffolds [17]. *Bradi1g29680*, *Bradi1g29690*, and *Brad1g29720* identified scaffold25403 (333kb), scaffold96474 (61kb), and scaffold5474 (44kb), respectively. Five SSR polymorphic markers *WGGC4659*, *WGGC4660*, *WGGC4661*, *WGGC4662*, and *WGGC4663* were developed from these scaffolds (Table 2, Fig. 2, Fig. 3). None of the scaffolds in the *T. urartu* genome matched *Bradi1g29700* and *Bradi1g29710*.

In addition, *WGGC4663* was used to screen the Chinese Spring 7AL-specific BAC library. The BAC Contig841 containing 12 BACs was identified and chosen for sequencing (Fig. 2; Keeble-Gagnere, manuscript in preparation). The 454 reads of 3 barcoded BAC pools were assembled into a contig spanning about 359.7 kb (GenBank accession No. KJ782373). Five additional polymorphic SSR markers *WGGC4664*, *WGGC4665*, *WGGC4666*, *WGGC4667*, and *WGGC4668* were developed from sequence contig841 and added to the high-resolution genetic map (Table 2, Fig. 2, Fig. 3). *WGGC4664*, *WGGC4665*, and *WGGC4668* were co-segregated, and 0.04 cM closer to *MlIW172* than *WGGC4663*. Moreover, *WGGC4667* and *WGGC4666* were 0.04 cM and 0.08 cM apart from *MlIW172* than *WGGC4663*, respectively (Fig. 2, Fig. 3). Therefore, the *MlIW172* was delineated to a 0.48 cM interval by *WGGC4664* (*WGGC4665*, *WGGC4668*) and *WGGC4659* (Fig. 2).

## Discussion

### Comparison of *MlIW172* with other *Pm* genes on 7AL

Our molecular marker analyses have revealed a dominant powdery mildew resistance locus, *MlIW172*, located on chromosome 7AL of *T. dicoccoides*. A number of powdery mildew resistance loci have been mapped to the same or nearby region. *Pm1* was the first reported powdery mildew resistance gene from common wheat cultivar Axminster and localized to 7AL arm [51], and subsequently, five *Pm1* alleles (*Pm1a*-*Pm1e*) have been described for the locus [3], [36], [46]. Genetic mapping analyses have shown that *Pm1* co-segregates with the *Xcdo347* and *PSR680* RFLP markers [37], and is 0.3 cM proximal to the *Xgwm344* SSR marker [46]. *MlIW172* co-segregates with the *PSR680* derived STS marker *MAG2185* in the preliminary genetic linkage map (Fig. 1B) and maps to a region flanked by *Xwmc525* and *Xgmw344*. Different powdery mildew reactions were observed between the *MlIW172* and *Pm1a* and *Pm1c* alleles after inoculation with 15 Chinese *Bgt* isolates (Table S1). Since none of the identified *Pm1* alleles was originated from wild emmer [3], the *MlIW172* is most likely a new allele of the *Pm1* locus.

**Figure 4:**
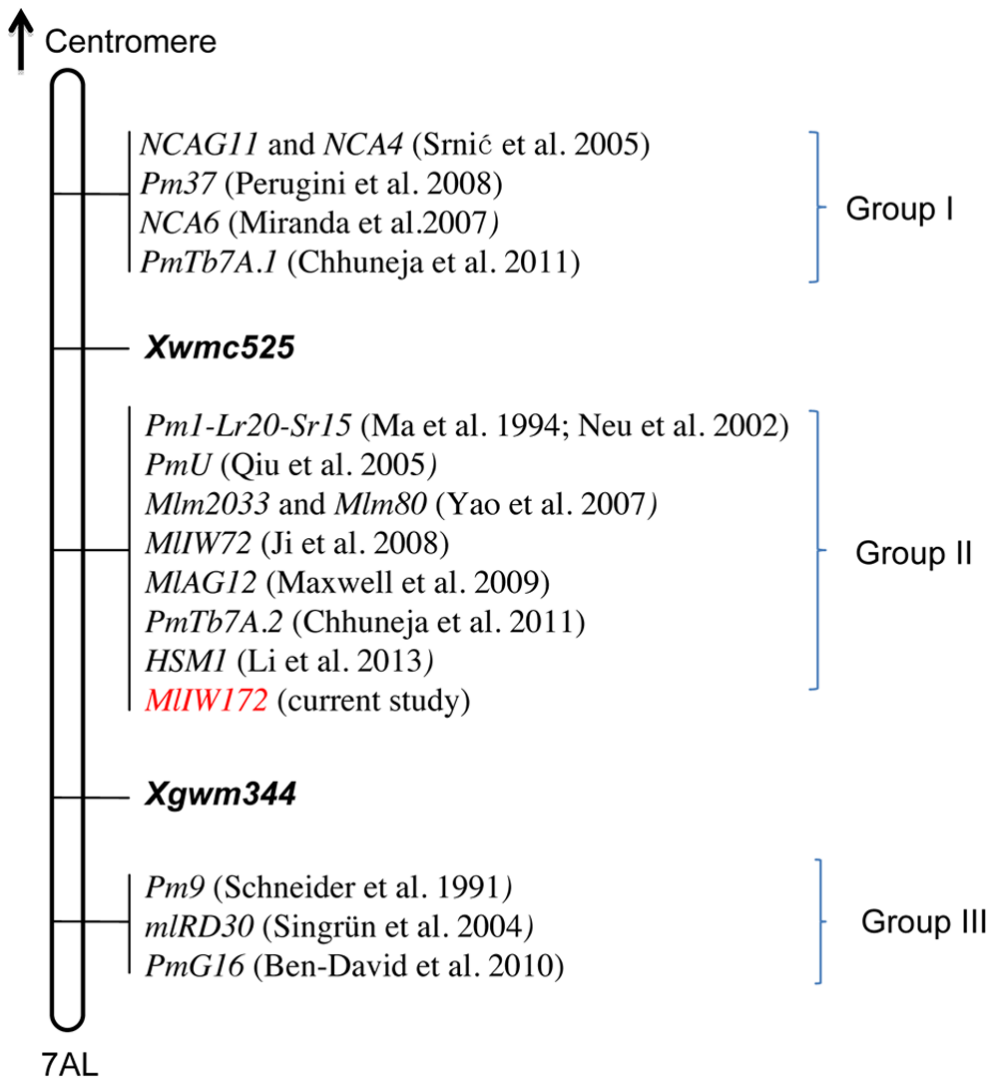
Integrative views of the *MlIW172* gene loci with other previously published *Pm* genes on chromosome arm 7AL. The loci can be classified into three groups (Group I; Group II; Group III) based on their order and genetic distance to markers *Xwmc525* and *Xgwm344*.

*Xwmc525* and *Xgwm344* are markers linked to several powdery mildew resistance genes located on chromosome arm 7AL. Based on their chromosome positions and genetic distances to the markers *Xwmc525* and *Xgwm344*, the powdery mildew resistance genes presently reported on 7AL can be classified into three groups (Fig. 4). The first group is proximal to *Xwmc525*, and includes *NCA4* and *NCAG11* [52], *NCA6* [53], *PmTb7A.1* [54] and *Pm37* [55]. The second group is located in the *Xwmc525* - *Xgwm344* genetic interval. Members of this group include the *Pm1* [36], [37], *Mlm2033* and *Mlm80* from *T. monococcum* [35], *PmTb7A.2* from *T. boeoticum* [54], *PmU* from *T. urartu* [56], *MlAG1*2 from *T. timopheevii* [57], as well as *MlIW72* [58], *HSM1* [10], and *MlIW172* (current study) from wild emmer. The *MlIW72* was identified from wild emmer IW72 collected in Kokhav Hashahar, Israel [58]. The IW172 (G-797-M) was a different collection from Israel. Although IW172 and IW72 were collected independently by different collectors, the possibility that the resistance genes *MlIW172* and *MlIW72* are identical cannot be excluded. The last group contains *PmG16* [6], and two recessive resistance genes *Pm9* [59] and *mlRD30* [60], which are located on the distal side of the *Xgwm344* marker on 7AL. Clearly, the distal 7AL bin appears to be rich in powdery mildew resistance genes. However, considering the influences of different mapping populations on the marker distance in this region, it is unclear whether the genes within one group represent a series of different loci, or are alleles at a single locus. Phytopathology test and allelism analyses in future should be able to clarify the relationships of these genes. The map-based cloning of one or more of the genes could delineate the diversity and variation of the powdery mildew resistance genes/alleles in this genomic region.

### Genetic and physical distance relationships in the *MlIW172* region

The majority of plant resistance genes (*R* genes) are members of large gene families. Understanding the evolution of *R* genes and the mechanisms underlying the evolution of novel *R* genes has become an important research field. Sequence rearrangement of *R* genes in multigene families through recombination can lead to the generation of novel *R* gene specificities. However, recombination in *R* gene regions can be complicated. Although most *R* genes are localized in high recombination regions [30], resistance loci with recombination suppression have been reported [37]. Suppressed recombination can cause co-segregation (complete genetic linkage) of multiple markers that may not be physically closely linked. The genetic and physical mapping in the 7AL distal region allowed us to examine the ratio of physical to genetic distance in the *MlIW172* region based on the analyses of the overlapping BAC sequences of A genomes from Chinese Spring, Langdon and *T. urartu* scaffolds (Fig. 4). Although our BAC contigs and sequence scaffolds were from different A genomes and did not completely cover the *MlIW172* region, we obtained a total of ∼929 kb of sequences that were anchored to the genetic map (Fig. 2). These sequences have greatly facilitated the development of molecular markers to increase the marker density in the *MlIW172* region and have allowed us to examine recombination within this region. With a genetic distance of 0.12 cM between the *WGGC4666* and *WGGC4668* markers (Fig. 1, Fig. 2), the ratio of physical to genetic distance on the proximal side of *MlIW172* was about 1.07Mb/cM; whereas in the distal region between *WGGC4662* and *WGGC4659*, a ratio of 855.9kb/cM was calculated. However, a higher ratio of recombination (182.7kb/cM) was detected from *WGGC4656* to *XRGA-C6*. Overall, an average of 529.8kb/cM was found in the *MlIW172* genomic region. Hossain et al. [47] showed that the highest density of EST (or gene) loci was observed in bin 7AL16-0.86-0.90, where approximate 1 EST (or gene) mapped for every 362 kb. The distal region of 7AL (bin 0.94–0.99, 4% of the 7AL arm) is a gene-rich region with high recombination spanning 21 Mb with approximately 442 kb/cM [61]. Therefore, recombination in the *MlIW172* genomic region is comparable to the EST mapping data on 7AL. We did not note a severe suppression of recombination at the *Lr20-Sr15-Pm1* resistance locus on chromosome arm 7AL in hexaploid wheat [37].

### High collinearity of wheat and *Brachypodium* in the *MlIW172* genomic region

Comparative genomics analyses can be used to exploit the syntenic relationships among grass species for development of new markers linked to genes of interest by analyzing well-assembled genome sequence information available in model species [7], [62]. Nevertheless, many studies have reported poor levels of micro-colinearity between wheat and rice because of inversions, deletions, duplications, and other rearrangements [6], [7], [48], [63]. The isolation of the wheat disease resistance genes *Lr10*, *Lr21* and *Pm3* has also shown that wheat and rice have very limited colinearity in the relevant chromosomal regions. For example, the rice genome contains genes homologous to *Lr10* and *Pm3*, but at non-orthologous positions, indicating massive genomic rearrangements happened after the divergence of rice and wheat [64], [65]. *Brachypodium* is expected to show better synteny with wheat than rice and sorghum because it diverged more recently from the lineage leading to wheat [27].

In this study, we identified the *MlIW172* orthologous regions from rice, *Brachypodium*, and sorghum using marker sequence information mapped to the wheat *MlIW172* region. The 992-kb sequences of overlapping BACs and *T. urartu* scaffolds were also used for comparative genomics analysis. In general, wheat, rice, sorghum, and *Brachypodium* have good colinearity. One major difference within the genomic region is the *Brachypodium* contains a cluster of putative NBS-LRR genes, *Bradi1g29630*, *Bradi1g29640*, *Bradi1g29660* and *Bradi1g29670* that correspond to, or are very near to, the disease resistance cluster region including the *Lr20-Sr15-Pm1* on 7AL. These resistance-like genes in the RGA cluster in *Brachypodium* share homology to RFLP probe *PSR680* and markers *XRGA-C6* and *XRGA-B6*, but are not present in the corresponding genomic regions in rice and sorghum. This observation suggests that these resistance-like genes might exist in the ancestral pooideae specie after its divergence from rice. The presence of RGA clusters in the *Brachypodium* and wheat orthologous region further supports the utility of *Brachypodium* genome for comparative mapping of *Triticeae* species, particularly in those rapidly evolving disease resistance loci.

### *MlIW172* is located in an RGA-rich genomic region

Most of the cloned disease resistance genes in wheat, such as *Pm3b* [65], *Sr33* [66], *Lr1* [67], [68], *Lr10* [69], *Lr21* [70] belong to the nucleotide binding site leucine-rich repeats (NBS-LRR) *R* gene family. From the tetraploid wheat cv. Langdon BACs 133N2 and 865A17 sequences, we identified five NBS-LRR type RGAs. In the *T. urartu* scaffold25403, we predicted three successive RGAs from 124,590bp to 151,331bp that could constitute an additional RGA cluster. The large NBS-LRR gene family is often clustered within a resistance locus, so it is very difficult to isolate and clone genes eliciting a desired resistance function in the large polyploidy wheat genome without fine genetic map and good BAC-based physical map information.

In this study, we have delimited *MlIW172* to a 0.48 cM interval and linked to *WGGC4659* as closely as 0.04 cM. The *WGGC* markers identified in this study have allowed BAC pools from the developing Chinese Spring 7AL genome assembly to be located to the regions of the *MlIW172* locus and this represents a significant step toward positional cloning of *MlIW172*. Analysis of the Chinese Spring 7AL-BAC pool sequences has indicated a total of three BAC pools (ca 800 kb each) are present in the region immediately flanking the *MlIW172* locus, based on the presence of the *WGGC* sequences identified above. However, none of the identified BAC clones 133N2 and 865A17 (Langdon), the contig841 (Chinese Spring) and scaffold25403 (*T. urartu*) surrounding the region of *MlIW172* was from A genome of wild emmer. Recently, we have constructed a wild emmer BAC library with 10× coverage (Liu et al unpublished data) which could be used for chromosome walking to close the gap in the physical map using the new markers developed from the *MlIW172*-adjacent ends of contig841 and scaffold25403. Identification and sequencing of BAC clones spanning the uncovered region are the critical steps towards map based cloning of the *MlIW172* gene in wild emmer.

## Supporting Information

Table S1 **Phytopathology test of** ***MlIW172*** **and some known powdery mildew resistance genes to 15 Chinese** ***Bgt*** **isolates.**
(XLS)
